# Hybrid Repair of a Giant Postcatheterization Femoral Pseudoaneurysm Using Dual Long-Balloon Occlusion: A Case Report

**DOI:** 10.7759/cureus.112015

**Published:** 2026-07-03

**Authors:** Kazuyuki Ishibashi, Mamika Motokawa, Shariful Islam, Aslam Ahmed, Mohammad Moniruzzaman

**Affiliations:** 1 Cardiovascular Surgery, Ship International Hospital, Dhaka, BGD

**Keywords:** endovascular balloon occlusion, femoral artery pseudoaneurysm, giant pseudoaneurysm, hybrid vascular surgery, post-catheterization complication

## Abstract

Femoral artery pseudoaneurysm is a well-recognized complication of arterial catheterization. While ultrasound-guided thrombin injection and endovascular interventions are increasingly employed, open surgical repair remains mandatory for large, symptomatic, or complicated lesions. However, obtaining surgical vascular control can be extremely challenging in giant pseudoaneurysms that extend beyond the inguinal ligament and distort the femoral bifurcation anatomy.

A 60-year-old woman on maintenance hemodialysis presented with severe pain and tense swelling in her right groin two months after undergoing cardiac catheterization via a right femoral approach. Computed tomography revealed a giant pseudoaneurysm with a maximum diameter of 8 cm, extending proximally above the inguinal ligament and distally into the upper thigh. The pseudoaneurysm neck originated near the bifurcation of the superficial femoral artery (SFA) and profunda femoris artery (PFA). Due to signs of impending rupture, including excruciating pain and overlying skin discoloration, emergency surgery was indicated. Because conventional surgical exposure for proximal and distal clamping was deemed hazardous due to the massive size and anatomical complexity of the lesion, a hybrid approach was adopted. Through a contralateral femoral approach, dual-balloon occlusion of both the SFA and PFA was performed to control systemic inflow and retrograde collateral backflow. Immediate surgical incision of the pseudoaneurysm sac under a completely bloodless operative field allowed for precise identification and direct suture closure of the neck. The patient's postoperative course was uneventful, and she was discharged on postoperative day 3. At the two-month outpatient follow-up, she remained asymptomatic with no clinical evidence of recurrent pseudoaneurysm or wound-related complications.

Dual-balloon occlusion combined with open surgical repair represents a highly effective and safe hybrid strategy for treating giant femoral pseudoaneurysms when conventional vascular control is technically demanding or unsafe. This technique provides excellent visualization, facilitates precise neck closure, and minimizes surgical invasiveness in anatomically complex lesions.

## Introduction

In recent years, various vascular closure devices and dedicated hemostatic compression systems have been developed to reduce access-site complications associated with catheter-based diagnostic and therapeutic procedures [1]. Nevertheless, manual compression continues to be widely employed in many resource-limited settings.

Femoral artery pseudoaneurysm is a well-recognized complication following arterial catheterization [2]. Surgical repair is generally indicated for large, symptomatic, expanding, or complicated pseudoaneurysms. Standard surgical management involves obtaining proximal and distal vascular control, followed by identification and closure of the pseudoaneurysm neck. In contrast, selected small pseudoaneurysms with favorable anatomy can be treated using less invasive techniques such as ultrasound-guided thrombin injection [3]. However, giant pseudoaneurysms extending beyond the inguinal ligament frequently preclude both percutaneous treatment and conventional vascular control, presenting a significant technical challenge.

Here, we report a case of a giant postcatheterization femoral pseudoaneurysm extending above the inguinal ligament, in which conventional surgical vascular control was deemed hazardous. Successful repair was achieved using dual-balloon occlusion of the superficial femoral artery (SFA) and the profunda femoris artery (PFA) via a contralateral endovascular approach, which provided a bloodless operative field for secure suture closure of the pseudoaneurysm neck.

This case highlights the feasibility and utility of a hybrid endovascular-open surgical strategy for managing complex femoral pseudoaneurysms when conventional vascular control is difficult or unsafe.

## Case presentation

A 60-year-old woman presented to our hospital with a primary complaint of severe pain and swelling in her right groin. Her medical history was significant for chronic kidney disease, for which she was receiving maintenance hemodialysis twice a week.

Two months prior to presentation, she had undergone cardiac catheterization via a right femoral approach at another institution. On the day following the procedure, after returning home, she noticed gradual swelling in her right groin. Although she consistently complained of pain at the access site during every hemodialysis session, she was managed with clinical observation by her local physician. Over the course of two months, the pain worsened to the extent that it severely limited her physical mobility, prompting her to seek medical attention at our facility. On admission, her height was 155 cm, weight was 48 kg, body temperature was 36.7°C, heart rate was 83 beats/min, SpO₂ was 97% on room air, and blood pressure was elevated at 180/100 mmHg.

Physical examination revealed a tense, pulsatile mass in the right groin, extending proximally above the inguinal ligament and distally to the upper third of the thigh (Figure 1). Laboratory findings were as follows: hemoglobin, 8.8 g/dL (normal: 13.5 ± 2 g/dL); white blood cell count, 6,530/μL (4.0-11.0 × 10³/μL); platelet count, 290,000/μL (150-450 × 10³/μL); C-reactive protein, 24 mg/L (<10 mg/L); and serum creatinine, 7.14 mg/dL (0.52-1.04 mg/dL) (Table 1). Although the C-reactive protein level was mildly elevated, the patient had no fever, leukocytosis, wound discharge, or other clinical signs of systemic infection. The inflammatory response was therefore considered to be secondary to the giant pseudoaneurysm and the surrounding organized hematoma. 

**Table 1:** Laboratory findings at admission. AST: aspartate aminotransferase; ALT: alanine aminotransferase; CRP: C-reactive protein.

Laboratory test	Hospital admission	Normal range
Hemoglobin (g/dL)	8.8	13.5 ± 1
Hematocrit (%)	27.4	41 ± 5
Total leukocyte count (10^3^/µL)	6.53	4.0-11.0
Neutrophils (%)	70	40-75
Lymphocytes (%)	17	20-50
Monocytes (%)	8	2.0-10
Eosinophils (%)	5	1.0-6.0
Basophils (%)	0	<1
Platelets (10^3^/µL)	290	150-450
Creatinine (mg/dL)	7.14	0.52-1.04
Serum sodium (mmol/L)	141	137-145
Serum potassium (mmol/L)	4	3.5-5.1
AST (U/L)	25	14-36
ALT (U/L)	21	<35
CRP (mg/L)	24	<10

**Figure 1:**
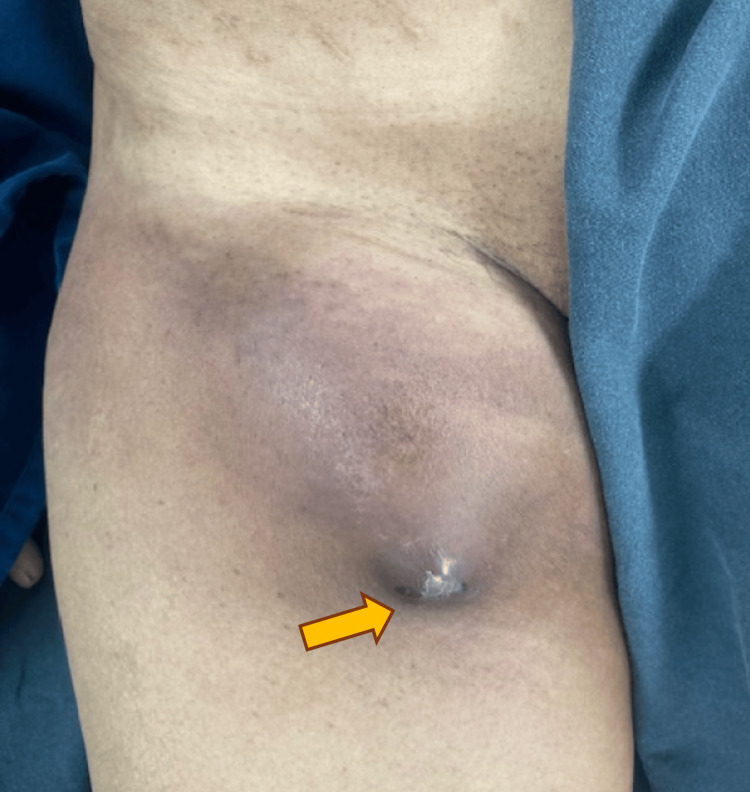
Preoperative macroscopic findings of the right groin. The massive pseudoaneurysm extended proximally beyond the inguinal ligament and distally into the upper third of the thigh. A focal area of dark-brown discoloration at the apex of the mass indicated impending rupture (arrow).

Computed tomography angiography (CTA) demonstrated a massive pseudoaneurysm with a maximum diameter of 8 cm (Figure 2D). The proximal end of the aneurysm extended above the inguinal ligament (Figure 2A), and the distal end reached the proximal third of the thigh (Figure 2D). The entry site (neck) of the pseudoaneurysm appeared to be located near the bifurcation of the SFA and PFA (Figure 2B,C). Due to excruciating pain and the development of reddish-brown skin discoloration over the mass, raising concern for impending rupture, emergency surgery was indicated. Given the massive size of the aneurysm, conventional surgical exposure for proximal and distal arterial clamping was anticipated to be extremely difficult. Therefore, we established a hybrid management strategy: endovascular balloon occlusion of both the SFA and PFA to control bleeding, followed by immediate surgical incision of the aneurysm to localize and close the entry site under a bloodless operative field. 

**Figure 2:**
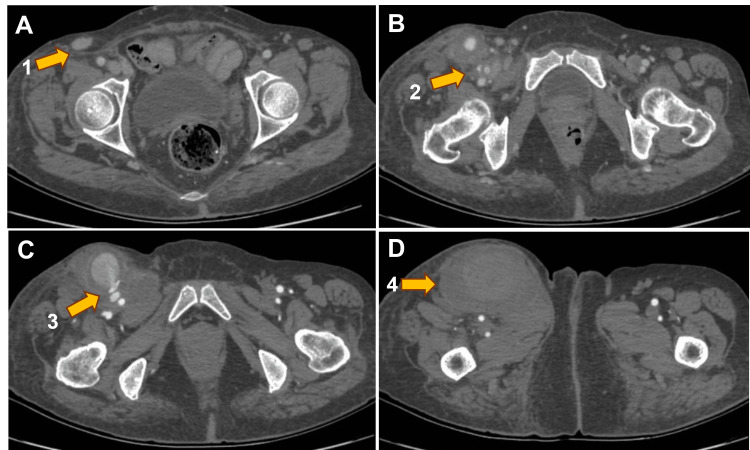
Preoperative computed tomography angiography (CTA) findings. (A) CTA image showing proximal extension of the pseudoaneurysm beyond the inguinal ligament (arrow 1). (B) CTA image showing the neck of the pseudoaneurysm located near the bifurcation of the superficial femoral artery (SFA) and profunda femoris artery (PFA) (arrow 2). (C) CTA image showing the giant pseudoaneurysm and its anatomical relationship to the femoral bifurcation (arrow 3). (D) CTA image showing the maximum diameter of the pseudoaneurysm (8 cm).

The procedure was performed under general anesthesia. Two 7-Fr sheaths (Glidesheath Slender™, Terumo, Tokyo, Japan) were inserted into the left common femoral artery. Following intravenous administration of an initial 3,000 units of unfractionated heparin, crossover access was obtained. Activated clotting time (ACT) was measured and found to be 180 seconds; therefore, an additional 2,000 units of unfractionated heparin were administered. Repeat ACT increased to 320 seconds, which was considered adequate for the procedure, and no further heparin was required. Using a 5-Fr multipurpose catheter (Outlook™, Terumo, Tokyo, Japan) and 0.035-inch guidewires (Radifocus™, Terumo, Tokyo, Japan), one wire was advanced into the contralateral (right) PFA and the other into the right SFA. Over these guidewires, two 6-Fr guiding sheaths (Destination™, Terumo, Tokyo, Japan) were advanced into the right common iliac artery. Preoperative CT demonstrated that the internal diameters of the right common femoral artery, superficial femoral artery, and profunda femoris artery were approximately 6 mm, 5 mm, and 5 mm, respectively. Balloon sizes were selected to achieve complete temporary arterial occlusion with slight oversizing; therefore, a 7-mm balloon was deployed from the CFA to the SFA, and a 6-mm balloon was positioned within the PFA. Right femoral angiography confirmed contrast extravasation near the SFA/PFA bifurcation. The neck of the pseudoaneurysm was identified near the origin of the PFA (Figure 3). To achieve complete inflow and backflow control, a 6 mm × 4 cm PTA balloon catheter (Fortrex™, Medtronic plc, Minneapolis, Minnesota) was inflated to occlude the PFA, and a 7 mm × 8 cm balloon catheter (Fortrex™, Medtronic plc) was inflated to occlude the segment from the common femoral artery (CFA) to the SFA (Figure 4). Both balloons were inflated to their nominal pressure (10 atm) to achieve temporary arterial occlusion. Completion angiography after balloon deflation demonstrated no evidence of arterial dissection, rupture, or other balloon-related vascular injury. 

**Figure 3:**
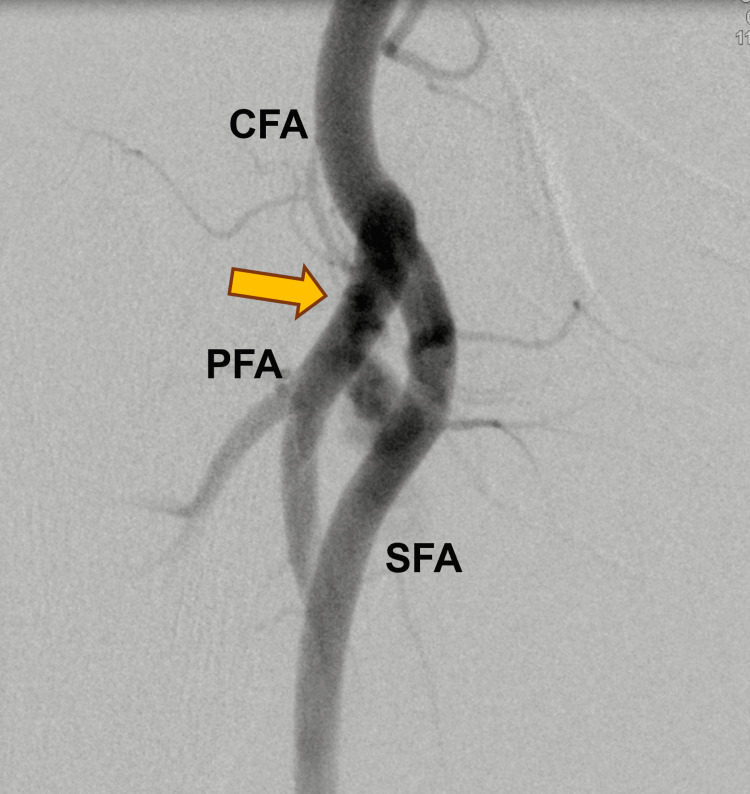
Preoperative digital subtraction angiography (DSA). The neck of the pseudoaneurysm originating near the ostium of the profunda femoris artery (PFA) is clearly visualized (arrow).

**Figure 4:**
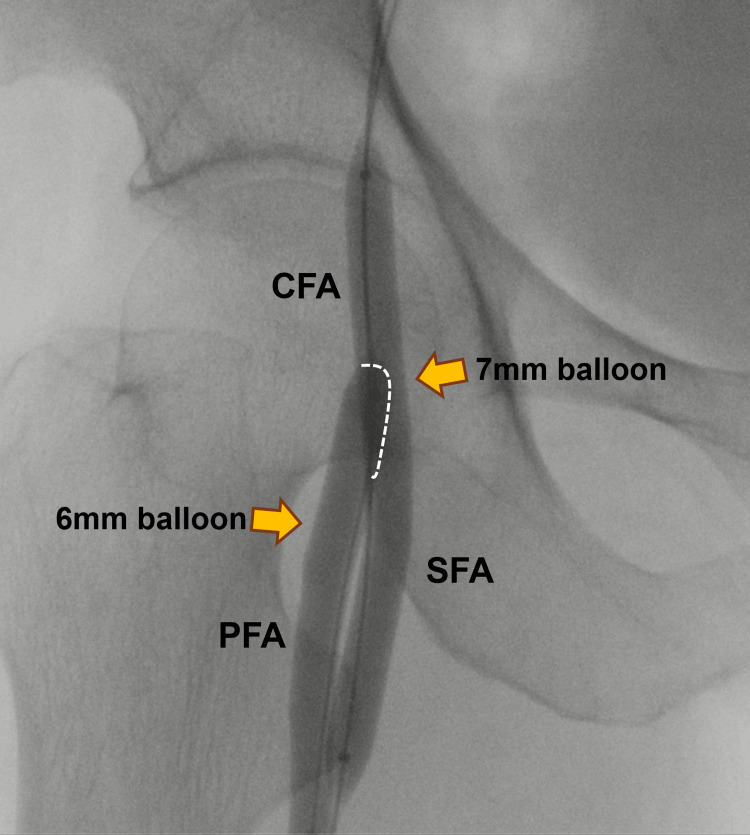
Fluoroscopic image showing intraoperative dual-balloon occlusion. A 7-mm balloon is deployed extending from the common femoral artery (CFA) to the superficial femoral artery (SFA), and a 6-mm balloon is positioned within the profunda femoris artery (PFA) to achieve complete inflow and outflow control and provide a bloodless operative field.

Subsequently, a longitudinal incision was made directly over the aneurysm. Although a transient gush of retained blood and hematoma occurred upon incision, an excellent, bloodless operative field was successfully achieved. We initially attempted to isolate and clamp the external iliac artery, SFA, and PFA surgically; however, dense inflammatory adhesions around the massive pseudoaneurysm rendered safe dissection impossible. As the pseudoaneurysm wall and organized thrombus were debrided, the entry site originating from the PFA was clearly visualized. The pseudoaneurysm neck was identified and directly closed above the PFA trunk using 5-0 polypropylene interrupted sutures (Figure 5). 

**Figure 5:**
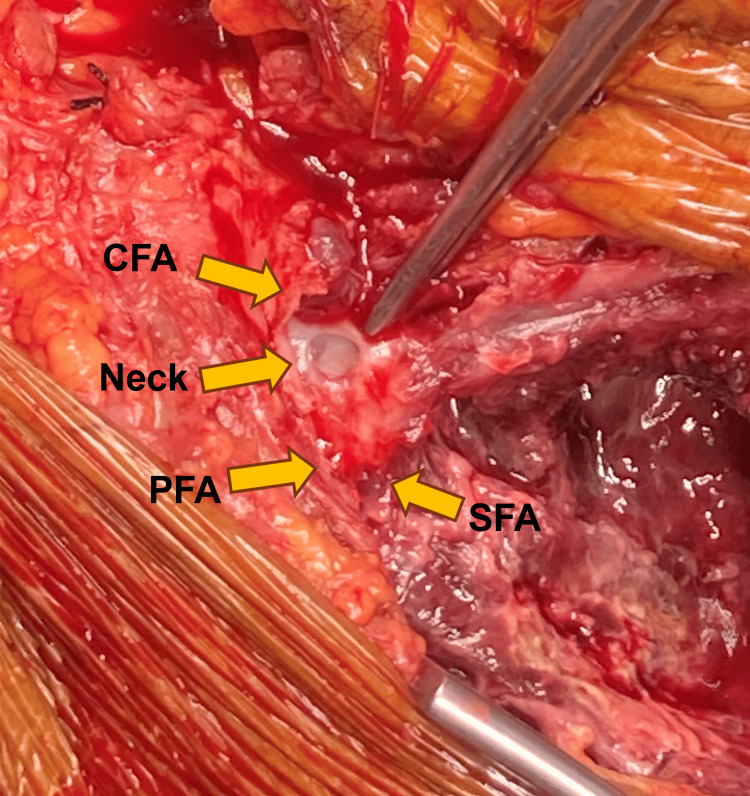
Intraoperative photograph after incision of the pseudoaneurysm. The pseudoaneurysm neck (arrow) is clearly identified under a bloodless operative field achieved by dual-balloon occlusion. The common femoral artery (CFA), superficial femoral artery (SFA), profunda femoris artery (PFA), and pseudoaneurysm neck (arrow) are labeled to facilitate identification of the anatomical landmarks.

After direct closure of the pseudoaneurysm neck with 5-0 polypropylene sutures and balloon deflation, intraoperative completion angiography was performed on the operating table. The angiogram confirmed complete exclusion of the pseudoaneurysm without contrast extravasation and preserved distal arterial flow (Figure 6). The total lower limb ischemic time, defined as the duration of balloon occlusion, was approximately 15 minutes. The wound was then closed after satisfactory hemostasis had been confirmed. Because the surrounding subcutaneous and muscular tissues were heavily infiltrated with blood, meticulous debridement and hemostasis were performed, and the wound was closed in three layers. The left femoral sheaths were removed, and the operation was concluded. The estimated intraoperative blood loss was approximately 20 mL. Although a large amount of organized hematoma was evacuated from the pseudoaneurysm sac, its volume and weight were not measured. A total of only 15 mL of iodinated contrast medium was required because the hybrid balloon-assisted technique relied primarily on fluoroscopic guidance and direct surgical visualization. No purulent fluid, necrotic tissue, or other gross findings suggestive of infection were observed intraoperatively. The patient's postoperative course was uneventful, and she was discharged on postoperative day 3. At the two-month follow-up visit, the patient remained free of groin pain or swelling, and no clinical findings suggestive of pseudoaneurysm recurrence or wound complications were observed.

**Figure 6:**
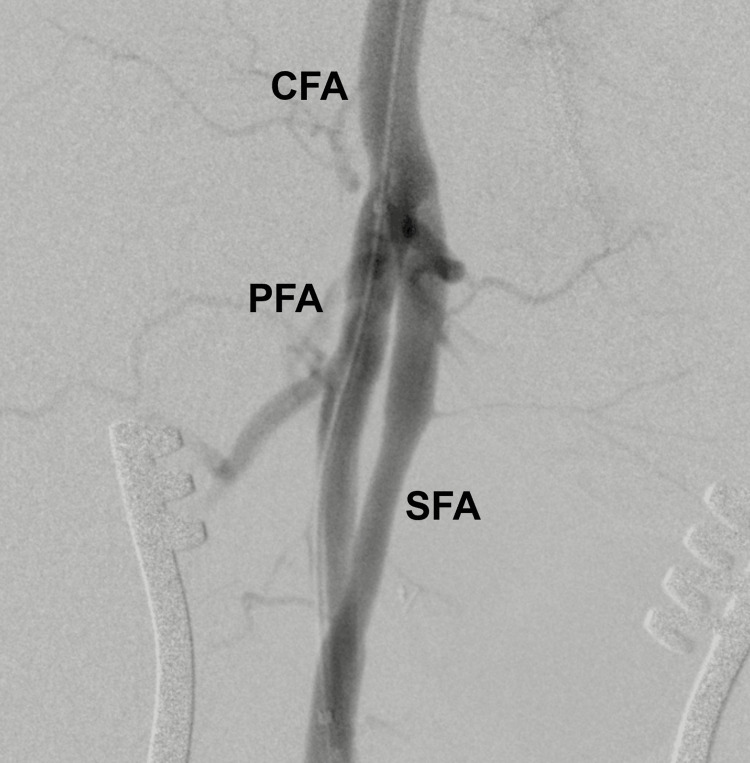
Intraoperative completion digital subtraction angiography (DSA). Intraoperative completion angiography obtained immediately after direct closure of the pseudoaneurysm neck and balloon deflation. No residual contrast extravasation is observed, and distal flow through the superficial femoral artery and profunda femoris artery is preserved.

## Discussion

Femoral artery pseudoaneurysm remains one of the most common vascular complications following femoral arterial catheterization. Although relatively uncommon overall, the reported incidence ranges from approximately 2.9% to 3.8%. Although its incidence has decreased with improvements in puncture techniques, vascular closure devices, and hemostatic systems, pseudoaneurysms continue to occur, particularly following femoral access. Management options include ultrasound-guided compression, thrombin injection, endovascular interventions, and open surgical repair. Among these, surgical repair remains the preferred treatment for symptomatic, expanding, infected, or ruptured pseudoaneurysms [2].

The present case had several unique features that made conventional surgical management particularly challenging. First, the pseudoaneurysm was exceptionally large, extending proximally beyond the inguinal ligament and distally into the upper thigh. Such extensive enlargement distorted the native anatomy and obscured the vascular landmarks required for safe proximal and distal arterial control. Furthermore, preoperative CTA demonstrated that the neck of the pseudoaneurysm originated near the bifurcation of the SFA and PFA. Therefore, standard vascular control would have required isolation of both branches in addition to proximal control of the common femoral or external iliac artery.

Second, the patient presented with severe pain, tense swelling, and overlying skin discoloration suggestive of impending rupture, necessitating emergency intervention. Because of the large size of the pseudoaneurysm and the extensive surrounding hematoma, ultrasound-guided thrombin injection was considered inappropriate due to the heightened risk of inadvertent thrombin migration and subsequent distal embolization.

Endovascular repair using a covered stent graft is another potential minimally invasive modality for treating femoral pseudoaneurysms [4]. However, this approach was considered unfeasible in the present case for two primary reasons. First, because the pseudoaneurysm neck was located precisely at the bifurcation of the SFA and PFA, deployment of a covered stent graft across the lesion would have inevitably covered and occluded one of these critical major branches. Second, the groin is a high-flexion zone; placing a covered stent graft across the hip joint carries a high long-term risk of stent fracture, deformation, or late occlusion. Consequently, open surgical repair under endovascular assistance was determined to be the safest and most durable option.

Some authors have reported hybrid approaches using balloon occlusion to facilitate the repair of difficult femoral pseudoaneurysms [5]. In a previously reported case involving a pseudoaneurysm that developed after stent placement extending from the external iliac artery to the SFA, short balloon catheters were used to achieve temporary vascular control [6]. However, such a strategy may not completely eliminate collateral inflow through neighboring branch vessels and may fail to provide a truly bloodless operative field.

The most critical technical aspect of the present case was the simultaneous deployment of two long balloon catheters: one positioned from the CFA to the SFA and the second deployed within the PFA. This configuration effectively minimized both antegrade arterial inflow and retrograde collateral backflow, thereby providing a nearly bloodless operative field. As a result, a completely bloodless operative field was obtained immediately upon opening the pseudoaneurysm sac. This allowed for precise identification of the entry site and secure suture closure, despite the distorted and complex anatomy around the femoral bifurcation. Appropriate balloon sizing is an important technical consideration. Because balloon oversizing and excessive inflation may increase the risk of arterial injury, balloon diameters were selected based on preoperative CTA measurements with only slight oversizing and inflated only to the manufacturer's nominal pressure. No balloon-related vascular injury was observed on completion angiography. The balloon occlusion time was limited to approximately 15 minutes. Although continuous distal limb perfusion monitoring was not performed, distal arterial flow was immediately restored after balloon deflation and confirmed by completion angiography, and no clinical signs of ischemic complications were observed postoperatively.

Another major advantage of this strategy was the avoidance of extensive surgical dissection. In giant pseudoaneurysms, dense inflammatory adhesions and hematoma formation often make the identification and taping of the external iliac artery, SFA, and PFA technically demanding and hazardous. Excessive dissection can significantly prolong operative time and increase the risks of iatrogenic arterial, venous, or nerve injury, as well as substantial blood loss [2]. In our patient, achieving proximal control of the external iliac artery would likely have required either retroperitoneal exposure or laparotomy because the pseudoaneurysm extended above the inguinal ligament. Given the patient's chronic renal failure and dependence on hemodialysis, minimizing surgical invasiveness and preserving future access sites were particularly desirable.

Several factors may have contributed to the remarkable enlargement of the pseudoaneurysm in the present case. The lesion remained untreated for approximately two months after catheterization, and the patient also had poorly controlled hypertension (180/100 mmHg) despite maintenance hemodialysis. Although the relative contribution of each factor cannot be determined, prolonged arterial pressure acting on an untreated arterial defect likely promoted progressive expansion of the pseudoaneurysm.

Although the patient's C-reactive protein level was mildly elevated preoperatively, neither the clinical presentation nor the intraoperative findings suggested an infected pseudoaneurysm. The inflammatory response was therefore attributed to chronic inflammation associated with the large pseudoaneurysm and surrounding organized hematoma.

The patient also had chronic kidney disease requiring maintenance hemodialysis, accompanied by anemia and severely impaired renal function. These comorbidities placed her at increased perioperative risk and further supported our strategy of minimizing surgical invasiveness. By avoiding extensive retroperitoneal dissection or laparotomy through the use of endovascular balloon-assisted vascular control, operative trauma and blood loss were minimized, which may have contributed to her uneventful postoperative recovery.

There are several limitations to this report. First, this is a single-case experience from a single institution treated by a single surgical team; therefore, the reproducibility and generalizability of this dual-balloon technique require further validation. Second, the current follow-up period is limited to two months. Although the patient remains clinically asymptomatic without evidence of recurrent pseudoaneurysm, mid- and long-term outcomes remain unknown. Third, postoperative imaging surveillance with duplex ultrasonography or computed tomography angiography was not performed because the patient remained clinically asymptomatic. Future studies incorporating routine imaging follow-up will be necessary to evaluate the durability of this technique.

In addition, endovascular balloon occlusion carries potential procedural risks, including iatrogenic vascular injury, such as arterial dissection or rupture caused by balloon overinflation, as well as distal thromboembolism during temporary arterial occlusion. Therefore, meticulous balloon sizing, careful inflation under fluoroscopic guidance, and minimization of balloon occlusion time are essential. Further cumulative studies are warranted to establish the long-term safety and reproducibility of this hybrid approach.

## Conclusions

In conclusion, this case suggests that dual-balloon occlusion may represent a promising hybrid strategy for the surgical management of giant femoral pseudoaneurysms in anatomically challenging situations. By providing temporary vascular control and a bloodless operative field, this technique may facilitate safe identification and closure of the pseudoaneurysm neck. Further experience and longer-term follow-up are necessary to confirm its safety and reproducibility.
